# Color and Translucency Stability of Three-Dimensional Printable Dental Materials for Crown and Bridge Restorations

**DOI:** 10.3390/ma14030650

**Published:** 2021-01-31

**Authors:** Jong-Eun Kim, Won-Huy Choi, Dasun Lee, Yooseok Shin, Sung-Ho Park, Byoung-Duck Roh, Dohyun Kim

**Affiliations:** 1 Department of Prosthodontics, Yonsei University College of Dentistry, 50-1 Yonsei-ro Seodaemun-gu, Seoul 03722, Korea; gomyou@yuhs.ac; 2 Department of Conservative Dentistry, Yonsei University College of Dentistry, 50-1 Yonsei-ro Seodaemun-gu, Seoul 03722, Korea; wonhuychoi@gmail.com (W.-H.C.); leedasun@yuhs.ac (D.L.); densys@yuhs.ac (Y.S.); sunghopark@yuhs.ac (S.-H.P.); operatys16@yuhs.ac (B.-D.R.)

**Keywords:** color stability, translucency, three-dimensional printing, dental material, crown and bridge

## Abstract

The purpose of this study was to examine and compare color and translucency stability of three-dimensional (3D) printable dental materials for crown and bridge restorations. Five different materials were investigated, and twelve disc-shaped specimens of two different thicknesses (1 and 2 mm) were prepared using a digital light processing 3D printer. Color measurements were made according to the CIELAB color scale (L*, a*, and b*) using a spectrophotometer 1 h, 1 day, 1 week, one month, and six months after post-curing of the materials, and the translucency parameter (TP) was calculated. The L*, a*, b*, and TP values were compared among the different materials and storage periods using repeated measures analysis of variance. Color and translucency changes of the specimens after the different storage periods were compared with 1 h measurements to determine whether they exceeded clinically perceivable thresholds. The L*, a*, b*, and TP values showed significant differences according to the storage periods, as well as among the materials. Until one month, some materials demonstrated distinct color differences, while others showed small color differences below a clinically perceivable threshold. The translucency differences were not clinically perceivable for any specimen. After six months, all specimens demonstrated large color changes, whereas the changes in translucency were relatively small. In conclusion, the color of 3D printable dental materials changed with time, and the differences varied with the materials used. On the contrary, the changes in translucency were small. Overall, the materials became darker, more yellowish, and more opaque after six months of water storage.

## 1. Introduction

In recent years, three-dimensional (3D) printing technology has been rapidly developed and widely utilized in various areas. In particular, in the dental field, 3D printing has become popular as an additive manufacturing method for dental restorations or laboratory products [1,2]. Moreover, it can be used in synergy with other digital technologies, such as computer-aided design/computer-aided manufacturing (CAD/CAM) or cone-beam computed tomography (CBCT). Currently, 3D printing is widely deployed in various dental treatment procedures, such as prosthodontic rehabilitation, dental implants, mandibular reconstructions, surgical and nonsurgical endodontics, and orthodontics [3,4,5,6]. 3D printing has made dental treatment procedures more accurate, efficient, and predictable, and it is gradually replacing traditional methods.

Currently, 3D printed dental restorations are mainly used as provisional or interim restorations for fixed prostheses, for example, as temporary crowns and bridges, as well as removable prostheses such as temporary dentures [7]. In many clinical situations, provisional restorations are used for a long time, up to several months, in the course of the dental treatment. They serve an important diagnostic role as functional and esthetic try-ins and as blueprints for the design of the definitive prosthesis [8]. Occlusion, tooth contours, and pontic designs developed in the provisional restoration can be considered as a reference for the final restoration [9]. When the provisional restoration needs to be used for a long time, esthetic concerns and demands of patients will be increased, especially in anterior restorations. The optical properties of the provisional restorations, as well as their stability over time, are critical issues in this respect, which clinicians should carefully consider.

The color of a material is generally described as a shade based on the Munsell color system, which consists of three primary color attributes—hue, lightness, and chroma [10]. In addition to color, other optical properties need to be accounted for, such as translucency, opacity, opalescence, iridescence, and fluorescence [11]. Among these, translucency is regarded as one of the most important factors influencing the esthetics of dental restorations [12]. Translucency is the ability of a layer of a colored substance to allow an underlying background to show through [13]. Incident light undergoes reflection, absorption, scattering, and transmission within the dental material, and translucency is determined by the interaction of these phenomena [14].

While there have been several studies of the color and translucency of dental resin-based composites (RBCs) for direct restoration as well as indirect dental ceramic materials [15,16,17,18,19], only limited information is available about those of 3D printable dental materials. Revilla-León et al. measured the color of 3D printable dental materials and compared them with those of conventional acrylic resin-based interim materials [20]. They included five different 3D printable materials in the study; however, they did not evaluate the translucency or the color stability of the materials. Shin et al. evaluated the color stability of two 3D printable materials and three CAD/CAM blocks [21]. Although they observed the specimens in various storage media, the storage period was limited to only a month, and the translucency was not evaluated. Thus, studies on the color and translucency of 3D printable dental materials as well as their stability over an extended period of time are needed.

Therefore, the purpose of this study was to examine and compare the color and translucency of 3D printable dental materials for crown and bridge restorations. Moreover, the stability of color and translucency of each material for up to 6 months was assessed. The null hypotheses were that there are no differences in the color and translucency among the 3D printable dental materials used in this study and that color and translucency do not change during the storage period.

## 2. Materials and Methods

### 2.1. Specimen Preparation

Five different 3D printable dental materials for crown and bridge restorations were investigated (DT-1 A2 and A3 (HA2 and HA3; Hephzibah, Incheon, Korea), NextDent C&B MFH N1 and NextDent C&B A3.5 (NN1 and NA3; NextDent, Soesterburg, The Netherlands), and DIOnavi C&B A3 (DA3; DIO Inc., Busan, Korea). The characteristics and compositions of each material are summarized in Table 1.

Twelve disc-shaped specimens, 10 mm in diameter, of two different thicknesses (1 and 2 mm) were prepared with each material. The specimens were designed using the Rhino 6 software (Robert McNeel and Associates, Seattle, WA, USA) and fabricated using a digital light processing (DLP) 3D printer (Veltz D2; Hephzibah, Incheon, Korea), according to the manufacturers’ instructions. The thickness of each printed layer was set to 100 μm. After cleaning the specimens with isopropyl alcohol, the post-curing process was carried out using a UV chamber (CureM D102H; SONA Global, Seoul, Korea) with a UV intensity of 220 μW·cm^−2^ for 20 min. The specimens were polished with 1200-grit silicon carbide paper underwater cooling, and a digital caliper (500–181; Mitutoyo, Tokyo, Japan) was used to measure their thickness with a precision of 0.05 mm. The surface of each specimen was visually inspected, and the specimen was rejected if there were any defects or irregularities. Each specimen was stored in distilled water at 37 °C under dark conditions during the experiment. The distilled water was replaced every week.

### 2.2. Measurement of Color and Translucency

The color measurements were performed according to the CIELAB color scale [22,23] using a spectrophotometer (CM-2600d; Minolta, Osaka, Japan) with a 3 mm aperture. L* indicates lightness (0 to 100), which relates to the physical intensity of a color, and a* and b* indicate the levels of red (+a*), green (−a*), yellow (+b*), and blue (−b*) (−60 to 60). The L*, a*, and b* values of each specimen were measured and recorded relative to the standard illuminant D65 against black (L** = 1.38, a** = 0.00, b** = 0.06) and white (L** = 94.44, a** = 0.26, b** = 1.69) reflectance standards (Spectralon; Labsphere, North Sutton, NH, USA) after 1 h, 1 day, 1 week, 1 month, and 6 months of storage after post-curing of the specimen. Each specimen was measured in triplicate. The translucency parameter (TP) of each specimen was obtained by calculating the color difference of the specimen against the black and white standards, according to the following Equation (1) [13]:

TP = [(L*_B_ − L*_W_)^2^ + (a*_B_ − a*_W_)^2^ + (b*_B_ − b*_W_)^2^]^1/2^, (1)

where L*_B_, a*_B_, and b*_B_ were measured against the black background and L*_W_, a*_W_, and b*_W_ against the white background.

### 2.3. Calculation of Color and Translucency Differences

The color differences (ΔE) in the CIELAB color space of each specimen at different times were calculated against the 1 h measurement using the following Equation (2) [24]:

ΔE = [(L*_x_ − L*_1hr_)^2^ + (a*_x_ − a*_1hr_)^2^ + (b*_x_ − b*_1hr_)^2^]^1/2^, (2)

where the L*, a*, and b* values were measured against the white standard. The subscript “x” refers to the storage period, and the subscript “1 h” refers to the values measured at 1 h. A value of ΔE ≥ 3.7 was used as the threshold for a clinically perceivable color difference [25].

The difference in TP (ΔTP) was calculated by the following Equation (3):

ΔTP = TP_x_ − TP_1hr_, (3)

where the subscript “x” refers to the storage period and the subscript “1 h” to the TP value at 1 h. A value of |ΔTP| ≥ 2.0 was used as the threshold for a clinically perceivable translucency difference [26].

### 2.4. Statistical Analysis

The color parameters measured against the white background (L*, a*, and b*) and TP values were compared among the different materials and storage periods using repeated measures analysis of variance (RM-ANOVA) followed by Tukey’s post hoc tests. All statistical analyses were performed at the 95% confidence level using the SPSS 23 software (IBM Corp., Somers, NY, USA). The color difference (ΔE) and translucency difference (ΔTP) of each specimen at each storage period against the 1 h measurement were examined to establish whether they exceeded clinically perceivable thresholds.

## 3. Results

### 3.1. Color

**Figure 1:**
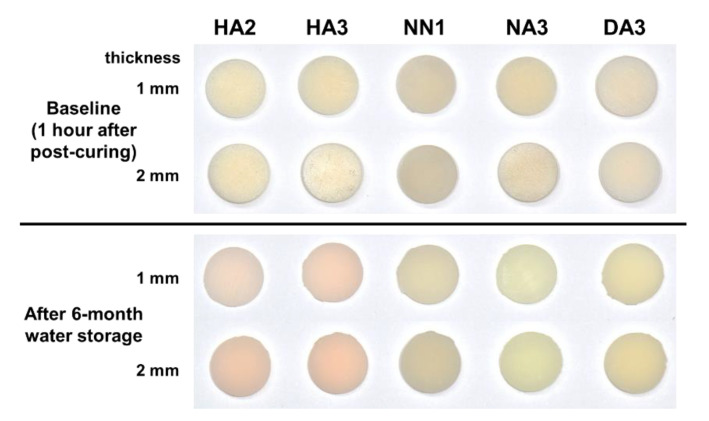
Photographs of the qualitative color changes of five 3D printable dental material specimens.

**Figure 2:**
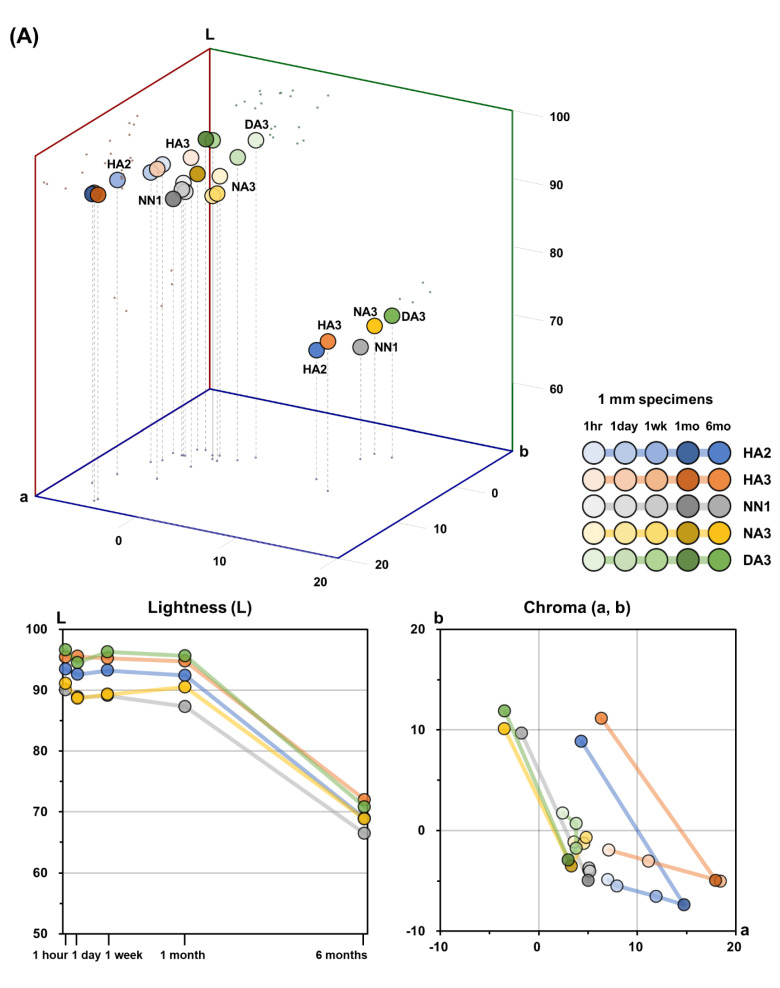

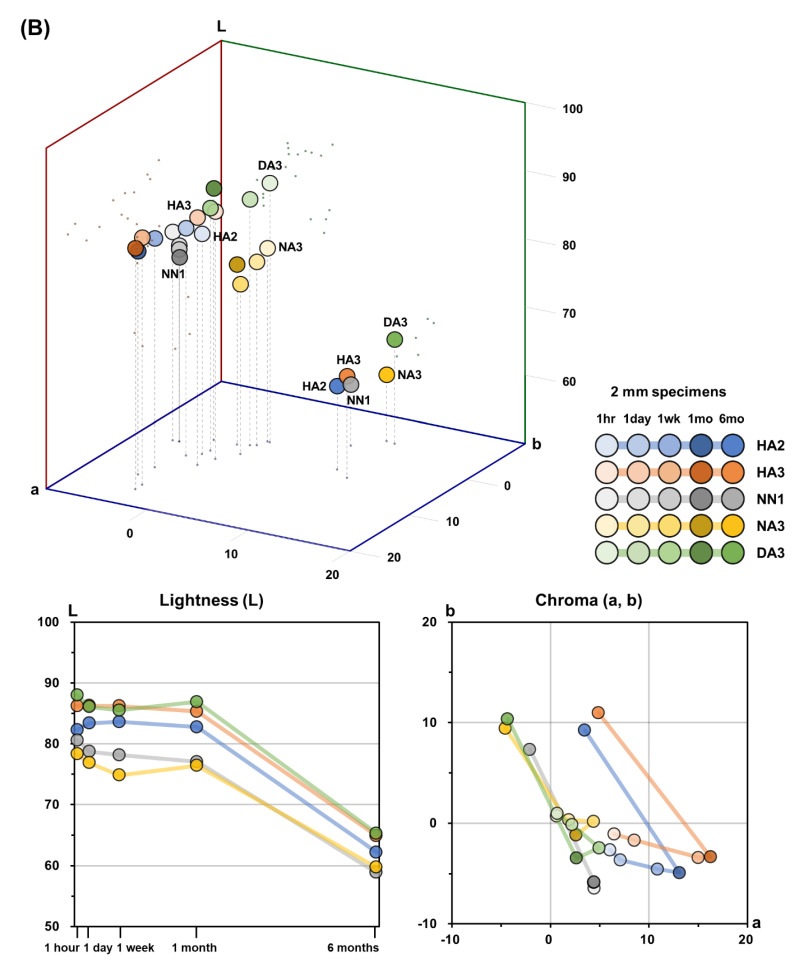
Mean color distribution of the 3D printable material specimens after different storage periods in the CIELAB color space (L*, a*, and b* coordinates). (**A**) 1 mm specimens. (**B**) 2 mm specimens.

Figure 1 presents qualitative color changes of the 3D printable dental material specimens after 6 months of water storage. The color distribution of the five 3D printable dental materials after each storage period in the CIELAB color space, and the corresponding L*, a*, and b* values, are presented in Table 2, Table 3 and Table 4 and Figure 2. The L*, a*, and b* values showed significant differences among the different storage periods, as well as among the different materials (*p* < 0.05).

For all materials, the 1 mm specimens showed higher L* values than the 2 mm specimens after all storage periods. For both 1 mm and 2 mm specimens, there was a significant difference in L* values among the five materials (*p* < 0.05), except between DA3 and HA3 and between NN1 and NA3. The L* values of HA3 after each storage period were higher than those of HA2, in both 1 mm and 2 mm specimens. No specific tendency was observed in the L* values of NN1 and NA3 according to storage periods. For both 1 mm and 2 mm specimens of all materials, no distinct change of L* values was observed until one month of storage. However, significant decreases in L* values were observed between 1 month and six months of storage for all materials.

There were significant differences in the * values among the five materials (*p* < 0.05), except between DA3 and NA3 in the 2 mm specimens. For all materials, the 1 mm specimens showed higher a* values than the 2 mm specimens after each storage period, except for DA3 after 1 week. For both 1 mm and 2 mm specimens, the a* values of HA3 and NN1 after each storage period were higher than those of HA2 and NA3, respectively. The a* value increased until one month for HA2 and HA3. For NN1, NA3, and DA3, there was no distinct change in a* values up to 1 month of storage. Decreases in a* values were observed from 1 month to 6 months of storage for all materials.

For both 1 mm and 2 mm specimens, there was a significant difference in b* values among the five materials (*p* < 0.05), except between HA3 and NN1 in 1 mm specimens. In both 1 mm and 2 mm specimens, the b* values of HA3 and NA3 after each storage period were higher than those of HA2 and NN1, respectively. For all materials, a decrease of the b* value until one month of storage and an increase in b* values from 1 month to 6 months of storage were observed.

**Figure 3:**
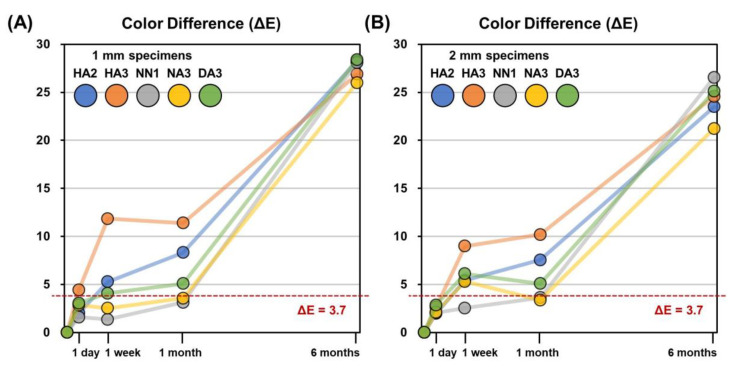
Mean color differences (ΔE) of the 3D printable material specimens after different storage periods compared to the 1 h color measurements. (**A**) 1 mm specimens. (**B**) 2 mm specimens.

The color differences (ΔE) of the specimens after each storage period, compared to the 1 h measurements, are presented in Figure 3; it was observed that the ΔE values varied depending on the material, but after six months, every group showed a clinically perceivable color difference, as indicated. In the first month, the HA3 specimens demonstrated the most distinct color differences; after 1 week, the ΔE values increased up to 11.8 and 9.0 in 1 mm and 2 mm specimens, respectively. In both 1 mm and 2 mm specimens, the HA2 and DA3 specimens showed clinically perceivable color differences after one week, but the ΔE values were lower than those of HA3. The color differences of the NN1 and NA3 specimens were below the clinically perceivable threshold during the first month. After six months, all specimens demonstrated quite large color differences compared with their original color.

### 3.2. Translucency

**Figure 4:**
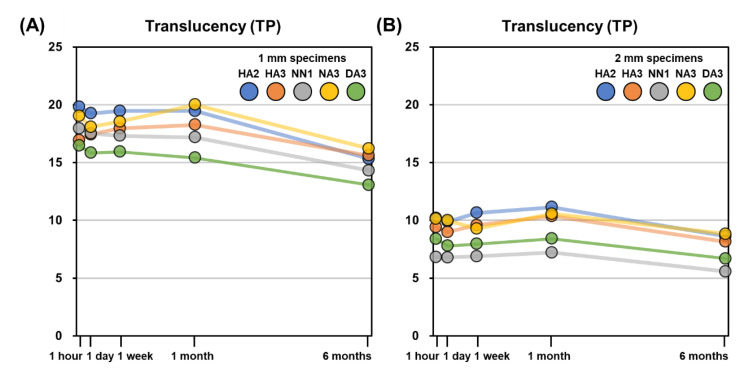
Mean translucency parameter (TP) of the 3D printable material specimens after different storage periods. (**A**) 1 mm specimens. (**B**) 2 mm specimens.

The TP values of the five 3D printable dental materials after each storage period are presented in Table 5 and Figure 4. In the 1 mm specimens, the TP value after 6 months of storage showed significant differences (*p* < 0.05), but there were no significant differences in the other storage periods. In 2 mm specimens, the TP values after 1 month and 6 months of storage were significantly different (*p* < 0.05), but there were no significant differences in other storage periods.

For all materials, the 1 mm specimens showed higher TP values than the 2 mm specimens. The TP values of 1 mm specimens were not significantly different among the materials (*p* > 0.05), except between DA3 and HA2 and between DA3 and NA3. For the 2 mm specimens, there were significant differences in TP values among all materials (*p* < 0.05), except between HA2 and HA3, HA2 and NA3, and HA3 and NA3. For the 1 mm specimens, there were no significant differences in TP values among the 1 h, 1 day, 1 week, and 1 month storage periods. Only the TP values after six months were significantly different from the measurement after the other storage periods (*p* < 0.05). For the 2 mm specimens, there was no significant difference between the TP values between 1 h and 1 week of storage. The TP values after one month and six months were significantly different from the measurement taken after the other storage periods (*p* < 0.05).

**Figure 5:**
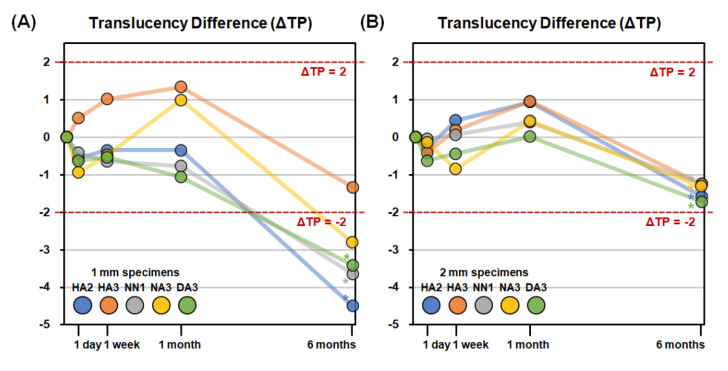
Mean translucency differences (ΔTP) of the specimens after different storage periods compared to the 1 h translucency measurements. (**A**) 1 mm specimens. (**B**) 2 mm specimens. Asterisk (*) indicates a statistically significant difference of TP value compared to the 1 h measurement.

The TP differences (ΔTP) after each storage period compared to the 1 h TP values are presented in Figure 5. The |ΔTP| values exceeded the threshold of 2.0 only for 1 mm specimens at six months of storage for the HA2, NN1, NA3, and DA3 materials. The changes in translucency were relatively small for all storage periods and were not clinically perceivable for any specimen up to 1 month. After six months, only 1 mm specimens demonstrated clinically perceivable translucency differences, specifically in the HA2, NN1, NA3, and DA3 specimens.

## 4. Discussion

The use of tooth-colored restorations fabricated by 3D printing is gradually increasing in dental clinics and laboratories. In the case of provisional restorations with tooth shades, the stability of the color and translucency is important, especially when the restoration needs to be used for a long time. In the present study, the color and translucency of five different 3D printable dental materials for crown and bridge restorations were evaluated and compared, and the stability of color and translucency after storage in distilled water for up to 6 months was assessed. As a result, it was observed that color and translucency varied among the different materials and changed depending on the storage period. Therefore, the null hypotheses of no differences in the color and translucency among the 3D printable dental materials used in this study and of no differences among storage periods were both rejected. Although a few studies have measured the color of 3D printable dental materials [20,21], there was no attempt to evaluate the translucency or their stability for an extended period. In this regard, the results of this study could be expected to provide relevant information for the use of 3D printable dental materials for crown and bridge restorations in dental laboratories and clinics.

The specimens were fabricated with two different thicknesses, 1 and 2 mm, as the thickness of dental crown and bridge restorations generally ranges from 1 to 2 mm [27]. However, it would become much thicker when the materials are used for interim prosthesis over implant abutments. For those purposes, further investigations, including specimens of various thicknesses and dimensions, would be needed.

The clinically perceivable threshold of color difference (ΔE) varies among studies [28]. The threshold criteria adopted in the present study, namely ΔE ≥ 3.7, are most widely applied when evaluating color differences [29,30,31]. Khashayar et al. [25] stated in their systematic review that this criterion refers to the study of Johnston et al. [32], which is the most commonly used reference as demonstrated by the citation ranking of the Web of Science. Additionally, the threshold values of ΔE ≥ 3.3 and ΔE ≥ 2.7 were suggested by Ruyter et al. [10] and Ragain and Johnston [33], respectively, and they have been applied in other studies as well. As there are still controversies on the gold standard of the threshold value, further investigations are needed for the clinically perceivable threshold of color difference to better reflect the clinical situations.

As shown in Figure 3, after one week of storage, the ΔE values exceeded the threshold for most materials, in both 1 mm and 2 mm specimens. From the 1 month period to the 6 month storage period, the ΔE values of every material drastically exceeded the threshold. These results imply that the color of the 3D printable materials used in this study began to become unstable after one week of storage, and by six months were completely unstable. In both 1 mm and 2 mm specimens, decreases of the L* value represented the largest contribution to the overall color difference, ΔE, from 1 month to 6 months of storage, as shown in Figure 2. In other words, the decrease in lightness was a major factor contributing to the color changes of 3D printable materials from 1 month to 6 months. On the contrary, during the first month of storage, changes of the a* and b* values were the main factors contributing to the overall color differences. Before one month, the decrease of the L* value was relatively smaller than between 1 and 6 months of storage. It should be noted that the trend of a* and b* values of all the materials used in this study were reversed after one month: until one month, the a* value increased and the b* value decreased; on the contrary, after one month of storage, the a* value decreased and the b* value increased. That is, materials became more reddish and bluish before one month, while after one month, they became more greenish and yellowish. These results imply that there are two phases of color change, a fast phase occurring during the first month and a slow phase occurring during six months. Moreover, at least two mechanisms of chemical reactions in the materials must be involved in these color changes.

In previous studies, factors that could possibly affect the color stability of dental RBCs and the rates of color change were evaluated. Light-cured RBCs are mainly comprised of matrix and filler, and they start to polymerize by the action of photoinitiators. The resulting polymer network is characterized by chemical interactions of esters, urethanes, amides, hydrogen bonds, and van der Waals forces [34]. Methacrylate derivatives are major constituents of the matrix of RBCs, and the ester bonds in methacrylates can be attacked by water, resulting in hydrolysis. The hydrolysis process of methacrylates is slow in neutral conditions, such as the distilled water used in our study. However, it needs to be considered because as the structure is gradually degraded and swelled with water sorption, unreacted monomers and degradation byproducts diffuse out more easily [35]. Another factor that should be taken into account is the polymerization rate, which is related to the concentration of residual monomers. Previous studies stated that the polymerization rate of 3D printable materials is relatively slow, even after the post-curing process [7,36]. Increasing residual monomers are related to surface softening as a result of the worsening surface integrity, increasing water sorption, and hydrolysis [37,38]. Berli et al. [39] reported that the water sorption of 3D-printed RBCs was actually higher than that of pressed and milled RBCs. Inorganic fillers may contribute to the stability of the materials as well. Fillers are used in dental RBCs for their mechanical properties, and they also affect the refractive indices of the RBCs. Fillers are coupled with organic substrates by coupling agents, the most popular being silanes. The oxane bonds between the filler and silanes are prone to hydrolysis due to their polarity [40]. During the degradation process of the resin matrix and coupling agents, changes in bulk microstructure are observed. Pores are formed via which monomers, degradation products, and additives are released [35]. Photoinitiators are known to be important in the color change of dental RBCs [41]. Phosphine oxide photoinitiators used in dental RBCs include diphenyl (2,4,6-trimethylbenzoyl) phosphine oxide (TPO) and bisphenyl (2,4,6-trimethylbenzoyl) phosphine oxide (BAPO) [42,43]. TPO and BAPO contribute to the yellow discoloration due to the production of colored peroxide by increased temperature during the polymerization process. A higher concentration of the photoinitiators results in a greater color change of the material [44]. Residual yellow discoloration after UV polymerization is greater in RBCs containing BAPO than in those containing TPO [45]. As the polymerization rate of 3D printable materials is slow, their color stability may be influenced by these photoinitiators.

The TP values of 1 mm specimens for all materials ranged from 15.4 to 20.0 until 1 month, and there were no significant differences in TP values according to the storage period for any material. Only the TP values after 6 months of storage significantly decreased from the 1 month values (*p* < 0.05) and ranged from 13.1 to 15.6. The TP values of 2 mm specimens were lower than those of 1 mm specimens for all materials and ranged from 6.8 to 11.1 until 1 month. The TP values after six months of storage significantly decreased from the 1 month period as well, but the decrease was much smaller than in 1 mm specimens (Figure 4). The absolute values of translucency differences, |ΔTP|, were relatively smaller than those of color differences, ΔE, for all storage periods. For the 1 mm specimens, the |ΔTP| values of all materials until one month were lower than 2.0, which is the clinically perceivable threshold of translucency difference according to Lee et al. [26]. After six months, the |ΔTP| values exceeded 2.0, except for HA3. All 2 mm specimens showed |ΔTP| values lower than 2.0 for all storage periods, regardless of the materials (Figure 5).

According to Yu et al. [46], the mean TP values of human enamel and dentin made into 1 mm specimens were 18.7 and 16.4, respectively. Pop-Ciutrila et al. [47] reported that the mean TP values of 2 mm specimens of human dentin were 6.85 on anterior teeth. Although measurements cannot be directly compared among studies, because of the varying experimental parameters, the TP values of the 3D printable materials used in the present study are reasonably similar to those of human teeth. Since the change of translucency was not perceivable in 2 mm specimens but was remarkable in 1 mm specimens, thinner restorations should be used more carefully in clinical situations.

The translucency of a dental material is related to the scattering of light. A mismatch of the refractive index between filler and matrix affects the transmission of light through the RBCs [48]. Ota et al. [49] stated that the refractive index is highly correlated with the TP value of RBC. Lee [50] reported that the TP value decreased as the amount of filler increased when the filler size was the same. As mentioned above, the degradation of matrix and coupling agent progresses slowly in water, which results in filler detachment and pore formation [35]. This process affects the refractory indices of the RBC, and therefore the translucency of the material.

This study has several limitations. First, although the components of 3D printable materials used in this study are roughly similar to conventional RBC materials, the exact chemical ingredient and content of the matrix, filler, and photoinitiators are unknown, being kept confidential by the manufacturers. Further analysis will be possible if more information on the constituents of 3D printable materials is released. Second, only one 3D printer and one post-curing chamber were used in the present study, with fixed curing time and light intensity, to prepare specimens using five different materials from four product lines. The post-curing method and time could affect the color of 3D printable materials. Kim et al. evaluated the effect of post-curing time on the mechanical and color properties of 3D printable dental materials [51]. They reported that the color changed as the post-curing time increased and that this would be mainly due to the photoinitiators, as stated earlier in the manuscript. According to the manufacturers’ instructions, the ideal combination of a 3D printer and post-curing machine leads to stable and optimal results. Moreover, the degree of conversion may vary depending on the curing time and other conditions. As post-curing parameters can affect the mechanical and chemical behaviors of 3D printable materials, the actual properties of the materials in clinical situations may vary. As optical properties of 3D printable materials have not been well-documented, further investigations should be performed under various post-curing conditions. Third, storage in distilled water cannot reflect the actual clinical conditions. In the present study, only distilled water was used for storage to standardize the preparation of the 6 month specimens. As it is known that the degradation of methacrylates is accelerated by changes in pH or the presence of enzymes [52], the effects of long-term storage in other solutions may be the subject of further studies.

Resin is the most widely used 3D printable material in dentistry. More recently, various types of 3D printable materials have been investigated for use in dentistry as well. Metals, such as cobalt-chrome alloy, can be used for fabricating fixed prostheses by 3D printing [53]. Silicone material has been used in medicine where the material is inserted into the body, as it is biologically inert; it can be used for maxillofacial prostheses [54]. A recent study reported that the direct 3D printing of meniscus implant with silicone material was successful [55], and the possibility that it may be utilized for dental implants is rising as well [56]. Further research related to these 3D printable materials should be undertaken.

## 5. Conclusions

Within the limitations of this study, the color of 3D printable dental materials for crown and bridge restorations changed with the passage of time after post-curing, and the differences varied with the materials used. On the contrary, the changes in translucency were relatively minor in all materials. Overall, the 3D printable dental materials became darker, more yellowish, and more opaque after six months of water storage.

## Figures and Tables

**Table 1:** 3D printable dental materials for crown and bridge restorations used in this study.

Code	Product Name	Shade	Manufacturer	Component *
HA2 HA3	DT-1	A2 A3	Hephzibah, Incheon, South Korea	Urethane acrylate, silicon oxides, pigments
NN1	NextDent C&B MFH	N1	NextDent, Soesterburg, Netherlands	Methacrylic oligomers, methacrylate monomer, inorganic filler, phosphine oxides, pigments
NA3	NextDent C&B	A3.5	NextDent, Soesterburg, Netherlands	> 90% methacrylic oligomers, methacrylate monomer, < 3% phosphine oxides, pigments
DA3	DIOnavi C&B	A3	DIO Inc, Busan, South Korea	> 90% methacrylic oligomers, < 10% phosphine oxides, pigments

* From the manufacturers’ instructions.

**Table 2:** Mean L* values of the 3D printable material specimens after different storage periods.

Specimen Thickness	Storage Period	HA2			HA3			NN1			NA3			DA3		
		Mean	SD		Mean	SD		Mean	SD		Mean	SD		Mean	SD	
1 mm	1 hour	93.44	2.17	a	95.41	2.13	a	90.02	1.95	a	91.07	1.81	a	96.62	2.46	a
	1 day	92.58	2.08	a	95.53	2.26	a	88.82	2.55	ab	88.65	2.24	a	94.53	2.81	a
	1 week	93.23	2.09	a	95.24	2.07	a	89.08	2.00	ab	89.28	2.15	a	96.28	2.46	a
	1 month	92.41	1.87	a	94.76	2.26	a	87.30	2.31	b	90.50	3.15	a	95.63	2.28	a
	6 months	68.92	1.88	b	71.97	1.62	b	66.44	1.67	c	68.79	1.84	b	70.79	1.97	b
2 mm	1 hour	82.29	2.03	a	86.24	1.88	a	80.59	0.86	a	78.34	1.42	a	88.01	1.39	a
	1 day	83.37	1.81	a	86.27	1.43	a	78.71	1.07	b	76.87	1.49	ab	86.06	1.11	bc
	1 week	83.60	1.77	a	86.22	1.06	a	78.15	0.87	b	74.85	1.90	c	85.52	1.00	c
	1 month	82.75	2.40	a	85.33	1.70	a	77.04	0.89	c	76.42	1.65	bc	86.91	1.05	ab
	6 months	62.20	1.83	b	64.90	1.08	b	58.89	0.62	d	59.73	1.09	d	65.30	0.81	d

Different letters denote significant differences among the specimens within each 3D printable material (*p* < 0.05).

**Table 3:** Mean a* values of the 3D printable material specimens after different storage periods.

Specimen Thickness	Storage Period	HA2			HA3			NN1			NA3			DA3		
		Mean	SD		Mean	SD		Mean	SD		Mean	SD		Mean	SD	
1 mm	1 hour	7.02	0.45	d	7.14	0.44	c	5.09	0.19	a	3.61	0.88	b	2.44	0.56	c
	1 day	7.95	0.70	c	11.13	1.11	b	5.11	0.25	a	4.58	0.65	a	3.79	0.62	a
	1 week	11.91	0.65	b	18.46	0.63	a	5.17	0.16	a	4.82	0.27	a	3.80	0.43	a
	1 month	14.74	0.72	a	17.97	1.17	a	5.02	0.21	a	3.31	0.33	b	2.99	0.33	b
	6 months	4.33	0.53	e	6.37	0.89	c	−1.76	0.06	b	−3.48	0.29	c	−3.43	0.22	d
2 mm	1 hour	6.03	0.28	d	6.46	0.32	d	4.47	0.12	a	0.64	0.67	d	0.69	0.43	d
	1 day	7.08	0.61	c	8.52	0.69	c	4.38	0.09	a	1.85	0.51	c	2.19	0.56	c
	1 week	10.86	0.87	b	14.99	0.76	b	4.40	0.07	a	4.37	0.14	a	4.93	0.31	a
	1 month	13.08	0.65	a	16.24	0.86	a	4.42	0.10	a	2.60	0.20	b	2.64	0.12	b
	6 months	3.49	0.47	e	4.87	0.64	e	−2.10	0.04	b	−4.57	0.11	e	−4.34	0.05	e

Different letters denote significant differences among the specimens within each 3D printable material (*p* < 0.05).

**Table 4:** Mean b* values of the 3D printable material specimens after different storage periods.

Specimen Thickness	Storage Period	HA2			HA3			NN1			NA3			DA3		
		Mean	SD		Mean	SD		Mean	SD		Mean	SD		Mean	SD	
1 mm	1 hour	−4.89	0.47	b	−1.97	0.88	b	−3.92	0.51	b	−1.16	0.87	b	1.74	1.11	b
	1 day	−5.50	0.50	c	−3.03	0.92	c	−3.72	0.65	b	−1.31	0.91	b	0.69	0.75	c
	1 week	−6.53	0.50	d	−5.02	0.88	d	−4.04	0.49	b	−0.72	0.74	b	−1.76	1.07	d
	1 month	−7.36	0.28	e	−4.95	0.67	d	−4.97	0.78	c	−3.54	1.06	c	−2.93	0.77	e
	6 months	8.83	0.44	a	11.14	0.44	a	9.69	0.31	a	10.10	0.26	a	11.86	0.63	a
2 mm	1 hour	−2.65	0.37	b	−1.08	0.59	b	−6.47	0.37	c	0.68	0.26	b	0.97	0.70	b
	1 day	−3.64	0.42	c	−1.68	0.43	c	−5.87	0.38	b	0.33	0.16	c	−0.15	0.58	c
	1 week	−4.55	0.43	d	−3.40	0.26	d	−5.85	0.29	b	0.16	0.41	c	−2.45	0.69	d
	1 month	−4.91	0.55	d	−3.33	0.16	d	−5.81	0.31	b	−1.18	0.28	d	−3.46	0.42	e
	6 months	9.23	0.29	a	10.98	0.26	a	7.34	0.21	a	9.44	0.32	a	10.37	0.31	a

Different letters denote significant differences among the specimens within each 3D printable material (*p* < 0.05).

**Table 5:** Mean translucency parameter (TP) of the 3D printable material specimens after different storage periods.

Specimen Thickness	Storage Period	HA2			HA3			NN1			NA3			DA3		
		Mean	SD		Mean	SD		Mean	SD		Mean	SD		Mean	SD	
1 mm	1 hour	19.81	2.09	a	16.93	1.69	ab	17.95	1.89	a	19.02	2.27	ab	16.46	2.21	a
	1 day	19.25	1.69	a	17.43	2.25	ab	17.53	2.09	a	18.08	3.35	ab	15.83	2.51	a
	1 week	19.47	1.60	a	17.95	1.86	a	17.31	2.13	a	18.54	2.83	ab	15.93	2.44	a
	1 month	19.47	1.61	a	18.27	1.86	a	17.18	2.35	a	20.01	3.52	a	15.41	2.03	ab
	6 months	15.32	1.57	b	15.60	1.54	b	14.30	1.36	b	16.21	2.39	b	13.06	1.65	b
2 mm	1 hour	10.20	1.55	ab	9.39	1.41	ab	6.82	0.48	a	10.13	0.85	ab	8.40	0.80	a
	1 day	9.89	1.52	ab	8.98	1.23	ab	6.78	0.53	a	10.00	0.78	ab	7.78	0.64	a
	1 week	10.65	1.75	a	9.58	1.01	ab	6.89	0.41	a	9.28	0.89	bc	7.96	0.63	a
	1 month	11.14	1.57	a	10.36	1.63	a	7.22	0.89	a	10.56	1.23	a	8.41	0.63	a
	6 months	8.63	1.25	b	8.14	1.03	b	5.57	0.57	b	8.82	0.87	c	6.68	0.46	b

Different letters denote significant differences among the specimens within each 3D printable material (*p* < 0.05).

## Data Availability

Data sharing is not applicable to this article.
